# Modulation of Genes and MicroRNAs in the Neurospheres of Glioblastoma Cell Lines U343 and T98G Induced by Ionizing Radiation and Temozolomide Therapy

**DOI:** 10.7759/cureus.32211

**Published:** 2022-12-05

**Authors:** Thiago L Da Costa Almeida, Andressa R Rodrigues, Múcio Cirino, Felipe A Trevisan, Fernanda Peria, Daniela Tirapelli, Carlos Gilberto Carlotti Jr

**Affiliations:** 1 Surgical and Anatomy, Ribeirão Preto Medical School, University of São Paulo, Ribeirão Preto, BRA; 2 Morphofunctional Laboratory and Integrated Practices, University of Franca, Franca, BRA; 3 Surgery and Anatomy, Ribeirão Preto Medical School, University of São Paulo, Ribeirão Preto, BRA; 4 Medical Practice, Medical School of Ribeirão Preto, Ribeirão Preto, BRA

**Keywords:** ionizing radiation, temozolomide, genes, mirna, neurospheres, glioblastoma

## Abstract

Introduction: Glioblastoma is the most prevalent primary malignant neoplasm of the central nervous system. It has increased its incidence, while the overall survival remains over 14 months.

Purpose: The purpose is to evaluate the expression of the genes EGFR, PTEN, MGMT, and IDH1/2, and microRNAs miR-181b, miR-145, miR-149, and miR-128a in adhered cells (AC) and neurospheres (NS) from cell lines (T98G and U343) submitted to temozolomide (TMZ) and ionizing radiation (IR).

Methods: T98G and U343 were treated with TMZ, IR, and TMZ+IR. The analysis of gene expression and miRNAs was performed using real-time PCR.

Results: This study demonstrated: a) an improvement in the expression of IDH1 after IR and TMZ + IR in the NS (T98G); b) an increase in the expression of MGMT in NS (T98G) in IR groups and TMZ + IR. The expression of miRNAs results as a) AC (U343) expressed more miR-181b after TMZ, IR, and TMZ + IR; and miR-128a improved after TMZ, IR, and TMZ + IR; b) NS (T98G) after TMZ + IR expressed: miR-181b; miR-149; miR-145 and miR-128a; c) NS (U343) after IR huge expressed miR-149 and miR-145.

Conclusion: IR was an independent and determining radioresistance factor in NS. However, we observed no complementarity action of oncomiRs regulation.

## Introduction

This study is a part of the author's doctoral thesis in which it sought to enumerate predictive and prognostic factors for a possible personalized intervention for glioblastoma, especially in view of the prospect of better results and lower toxicities [1]. Glioblastoma multiforme (GBM) is the main primary malignant neoplasm of the CNS. Its cellular and histological characteristics result in a median overall survival of only 15 to 18 months, with significant impairment to its patient´s quality of life. This characterizes its undifferentiated, aggressive and malignant profile, classifying GBM as grade IV regarding tumor cell differentiation, justifying being called “multiform” [2]. Recently it was found that the mutated gliomas in the HDI and TP53 genes and with codelection 1p19q gene had a more favorable prognosis [3]. In addition, it was evidenced that gliomas differ in their prognosis against the polymorphism of IDH mutations (mutated and non-mutated), codelection of 1p19q (Codel), and methylation of MGMT (G-CIMP high and G-CIMP low). In this sense, a new classification has been suggested among seven distinct molecular subgroups: G-CIMP-low, G-CIMP-high, Codel, Classic-like, Mesenchymal-like, LGm6-GBM, and PA-like [4].

Recent studies have shown that specific genes and microRNAs (miRNAs) can play key roles in GBM through regulatory pathways that drive and perpetuate their carcinogenesis [5,6]. Among the molecules with altered expression are cell membrane tyrosine kinases, such as epidermal growth factor receptor (EGFR); cytoplasmic enzymes of the mitogen-activated protein kinase (MAPK) signaling pathways; signaling enzymes PI3K (phosphatidyl inositol-3-kinase), and AKT (or PKB - protein kinase B), which show exacerbated self-performance [6].

This overactivation causes cellular hyperreplication through kinase-dependent cyclin and cyclin machinery, which prevents cell latency from phase G1 to phase S, perpetuating the tumor clone with carcinogenic mutation. However, tumor suppressors PTEN (phosphatase and tensin homolog), and IDH1/2 (isocitrate dehydrogenase), in addition to epigenetic regulation of the enzyme MGMT (O6-methylguanine-DNA methyltransferase) also present in this regulation [7].

The expression of 245 miRNAs implicated in GBM carcinogenesis is currently described [8,9]. While over 30% of genes are regulated by at least one miRNA, each miRNA can have hundreds or thousands of regulatory targets. Also, depending on the cellular context, the same miRNA may exhibit tumor suppressor and oncogenic activity [10]. In addition to observing differences between miRNA expression profiles in normal and tumor tissue, changes in the pattern of miRNA expression induced by radiotherapy (RT) and chemotherapy (QT) [4]. Among the existing miRNA population, miR-181b, miR-145, miR-149, and miR-128a stand out for their roles in GBM [11-13].

Cancer stem cells (CSC) may be involved in the origin and recurrence of GBM, thus being called GBM stem cells. This evidence signaled that a glioma can be formed and arise from GBM stem cells, which could remain quiescent until GBM carcinogenesis [14,15].

Regarding the therapeutic response, the GBM stem cells were radioresistant and chemoresistant, presenting a higher capacity to repair DNA damage when exposed to cellular stress [16]. From this, it was found that GBM stem cells have, as it were, a signature of specific genes and microRNAs and that, through studies of induction or interference in genes and miRNAs, they could participate in GBM therapy [17]. The neurospheres (NS) in the GBM, are found less than 1% viable in primary brain lineages. Despite the minimal representativeness, these cells have full heterogeneous differentiation and unlimited proliferation properties, being considered potentially responsible for GBM resistance and recurrence [18].

GBM is surgically incurable, favoring early or late locoregional recurrence. A better understanding of these therapeutic tools has consolidated combined surgical treatment (with maximum possible resection), followed by RT + TMZ and continuity of TMZ as a standard treatment against GBM [19]. In short, the current standard treatment for GBM does not result in a cure, as well as it allows its recurrence [19,20].

## Materials and methods

Cell culture 

U343MG and T98G - cell lines originally purchased from the American Type Culture Collection (ATCC) (Rockville, MD, USA) were kindly provided by Prof. James T. Rutka (The Arthur and Sonia Labatt Brain Tumour Research Centre, Canada); it was divided into suspension and adhesion culture and grown in 25 cm2 flasks (TPP®) with an ideal medium for cultivating brain CSCs, composed of Dulbecco's Modified Eagle's Medium/F12 medium (DMEM/F12, Gibco®), EGF (20ng/ml, Gibco®) and bFGF (20ng/ml, Gibco®) for the cells in suspension; Dulbecco's Modified Eagle's Medium/F12 medium (DMEM/F12, Gibco®) and 10% fetal bovine serum (FBS), for the cells to grow in attached monolayers on the flasks. All the cells were kept incubated at 37°C and 5% CO_2_ until they reached the cell confluence necessary (105 cells per culture flask).

Experimental subgroups 

In this study, we used 12 well plates and divided them into four experimental groups. In each subgroup (described below) 100,000 cells were placed, and all experiments were performed in triplicate.

Control Group

Cells were collected without any treatment.

TMZ Group

Temozolomide [340µM] (TEMODAL®, Schering-Plough, Turku, Finland) was dissolved in sterile water and filtered, and the drug remained in culture for 24 h. Cells were then washed with medium and replaced in culture for another 48 h.

IR Group

The radiation dose of 14 Gy was chosen on the basis of previous clonogenic survival experiments made by our group. 60Co source, dose rate of 2.0 Gy/min, Unit Gammatron S-80, Siemens, 1.25 MeV, HC-FMRP/USP, with a final dose of 14Gy.

TMZ+IR Group

Treatment with temozolomide following IR. This treatment sequence was based on the treatment protocol of patients with glioblastoma [4].

The cells of subgroups were collected and analyzed at 48 h after the treatments.

Cell viability

To assess cell viability, we used the exclusion test with Trypan Blue, a dye marker for dead cells. We gathered 50µL of Trypan Blue (0.4%). Cells were counted in a Neubauer counting chamber, wherein translucent cells were considered viable, and cells with blue staining were considered dead.

RNA isolation and real-time PCR 

Total RNA was extracted from cells of all experimental subgroups (30 min and 48h after temozolomide and ionizing radiation) using the Trizol reagent (Invitrogen, Carlsbad, CA, USA) according to the manufacturer's instructions. In order to verify the integrity of the RNA, each sample was subjected to electrophoresis on agarose gel 1% RNA, and using a spectrophotometer we determine the RNA concentration and purity (206/280 ratio) in the samples. To prepare the PCR, reverse transcription of RNA samples was performed using the High-Capacity cDNA kit (Applied Biosystems, USA).

Real-time PCR

The protocol for performing real-time PCR includes the synthesis of cDNA, which was amplified with quantitative real-time PCR (Q-PCR) using TaqMan Master Mix (Applied Biosystems) for the reaction of genes and microRNAs. The cDNA was amplified with quantitative real-time Polymerase Chain Reaction (q-PCR) using TaqMan Master Mix Applied Biosystems) for the reaction of microRNAs and gene reaction. The U6 gene was used as an endogenous control for the reaction of the microRNAs; however, for gene reaction, TBP and HPRT were used as endogenous control. The PCR conditions were preheating at 50° for 2 min, denaturation at 95° for 10 min, and 50 cycles of amplification and quantification (15s at 95°and 1 min at 60°). All reactions were carried out in duplicate and analyzed with the 7500 Sequence Detection System apparatus (Applied Biosystems). For the data analysis, we use the ABI-7500 SDS software [21].

Statistical analysis

Data concerning the microRNAs in all groups were analyzed statistically with a two-way ANOVA test followed by the Bonferroni post-test using the GraphPad Prism software (GraphPad Software, San Diego, CA, USA). The level of significance was set at p<0.05 for two-tailed tests.

## Results

Cell viability

**Figure 1:**
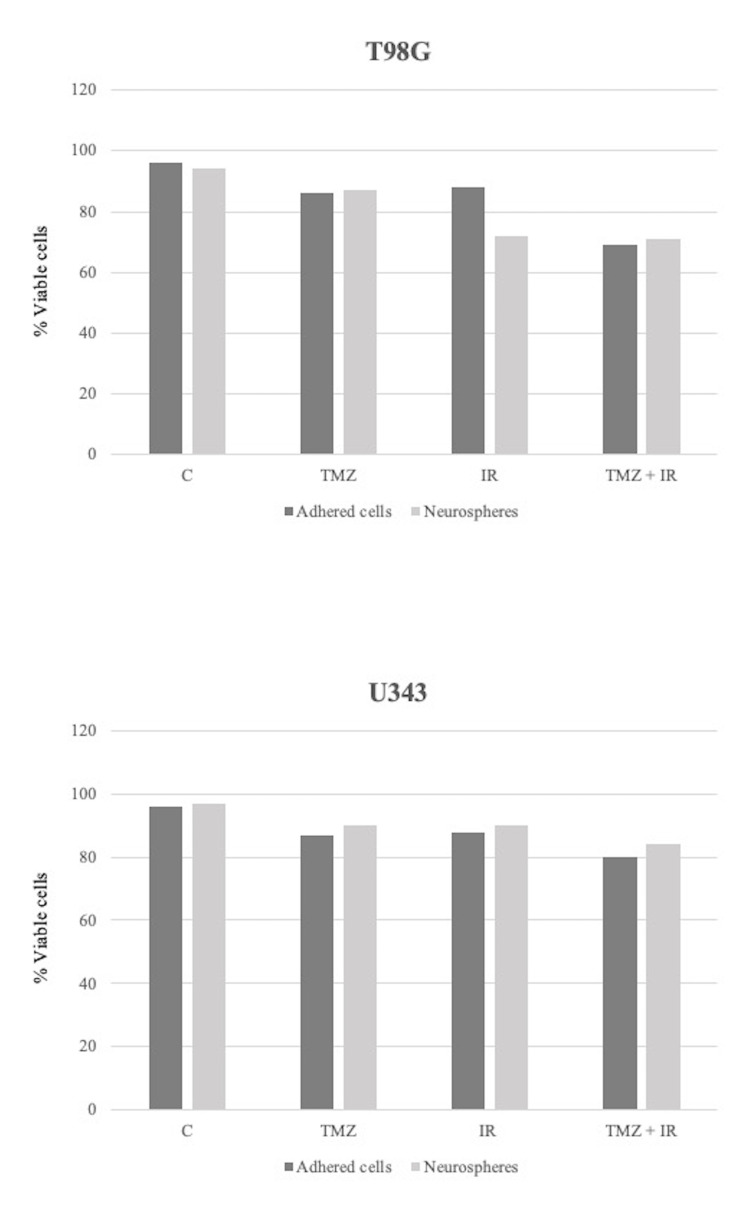
T98G and U343 cell viability in the control group and in the treatment modalities groups (TMZ, IR, TMZ + IR) in the studied subgroups: neurospheres and adhered cells.

From the graphs (Figure 1) , it is possible to observe differences in cell viability in the two lines analyzed (T98G and U343) and submitted to different treatments, however, there was no statistically significant difference between the groups.

Expression of IDH1 and IDH2 genes in samples submitted to different temozolomide and ionizing radiation treatments, isolated and associated

**Figure 2:**
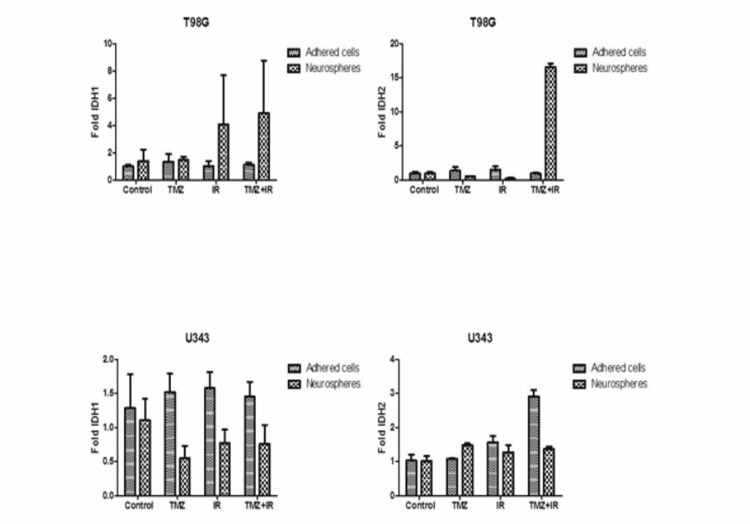
Expression of the IDH1 gene in the T98G cell line in the control group and in the groups submitted to treatment modalities (TMZ, IR, TMZ + IR) in the studied subgroups: neurospheres and adhered cells.

**Figure 3:**
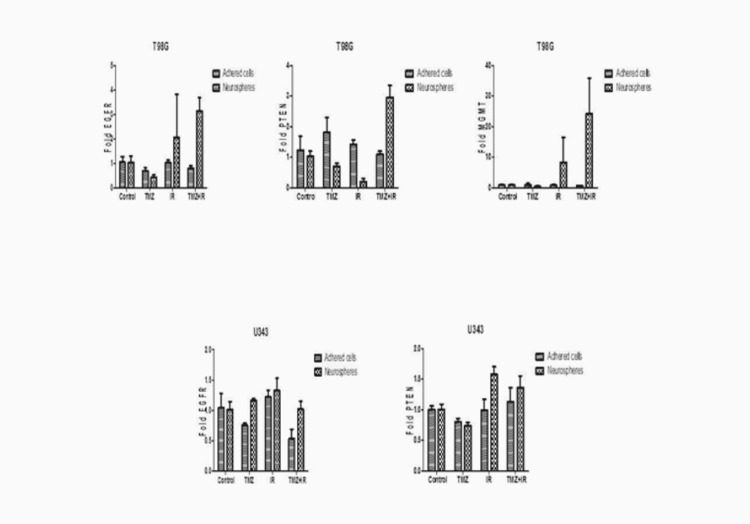
Expression of the EGFR, PTEN, and MGMT genes in the T98G and U343 cell lines in the control group and in the groups submitted to treatment modalities (TMZ, IR, TMZ + IR) in the studied subgroups: neurospheres and adhered cells.

In the T98G cell line, there was increased expression of the MGMT, IDH1/2, EGFR and PTEN genes and miR -181b, -145, -149 and -128a only when the NS were submitted to the TMZ + IR combined intervention modality (Figures 2, 3). These same miRNAs showed no expression in adhered cells, compared to the different treatments isolated, and even when associated, in this cell line. Thus, these miRNAs may be implicated with the increased expression of these genes in the NS, when submitted to TMZ + IR, by inhibiting their counter regulators.

**Figure 4:**
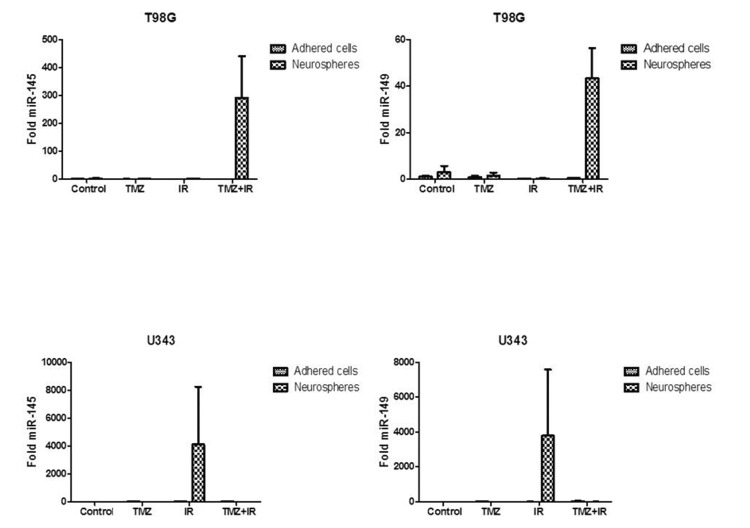
Expression of the microRNAs miR-145 and miR-149 in the T98G and U343 cell lines in the control group and in the groups submitted to treatment modalities (TMZ, IR, TMZ + IR) in the studied subgroups: neurospheres and adhered cells.

**Figure 5:**
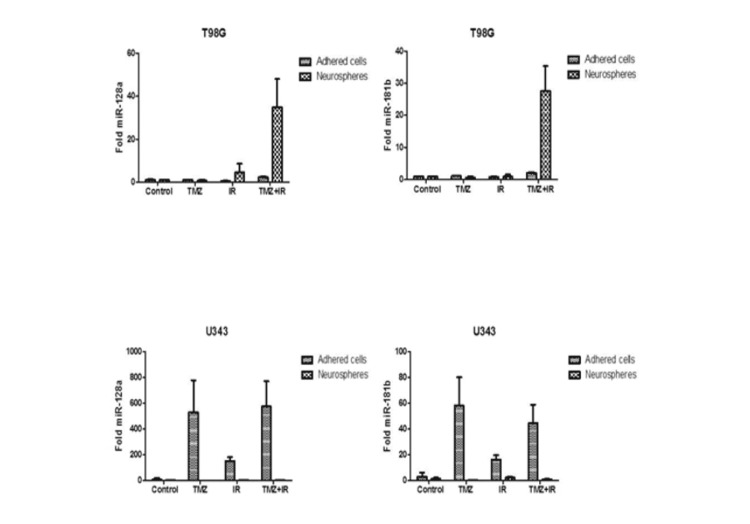
Expression of the microRNAs miR-128a and miR-181b in the T98G and U343 cell lines in the control group and in the groups submitted to treatment modalities (TMZ, IR, TMZ + IR) in the studied subgroups: neurospheres and adhered cells.

Noteworthy, there was a higher expression of the MGMT, EGFR and PTEN genes (Figure 3) and miRNAs -145, -149 and -128a (Figures 4, 5), which had their expression exuberant and potentiated when the NS were submitted to isolated IR, in the cell line U343. Combination treatment also increased these expressions, but to a lesser extent, perhaps by the addition of TMZ. In this context, IR is an independent and determining factor for radioresistance of NS, while it is an effective treatment for adhered cells. Otherwise, there was no downregulation complementarity action of the analyzed oncomiRs on the expression of the analyzed genes.

Increased expression of the genes and miRNAs analyzed was observed in both U343 and T98G cell lines, especially in NS, when submitted to IR and TMZ + IR, respectively. Thus, it is suggested that IR has a potential inducing effect of loss of quiescence in NS and stimulates radio and chemoresistance. In this sense, while the Stupp regimen is effective on tumor cells, it is hypothesized that TMZ-based sequential treatment, followed by TMZ + IR, could be better than the currently prevailing TMZ + IR, followed by TMZ, including GBM stem cells for treatment.

## Discussion

Lai et al. reported that GBM with mutated IHD1/2 has a better prognosis after standard treatment (TMZ + IR). In that regard, in the present study, the wild IDH1/2 gene was observed with hyperreactivity, after intervention with TMZ in adhered cells, while there was a greater hyperreactivity to stimulation by IR, especially in NS. Still in that sense, considering the participation of IDH1/2 in GBM neoangiogenesis, via the HIF-1a pathway and in the promotion of oncogenesis via the 2HG pathway, as evidenced by Verhaak, it may suggest that the action of IR on NS interrupts its quiescence, making it favorable to gliomagenesis and neoangiogenesis [21]. 

Stockhausen et al. stated that dedifferentiation (from differentiated cells - adhered to stem cells, neurospheres, in this case) reduces tumor proliferation and stabilizes neurospheres, signaling that EGFR blockade would increase the target on the GBM stem cells population, so it may suggest that TMZ intervention was effective on adhered cells, while stimulating the differentiation of NS in those submitted to IR [22]. Even O'rourke et al. and Li et al. argue that this process may explain the hypothesis of gliomagenesis recurrence from GBM stem cells after IR stimulation and, in particular, the promotion of its radioresistance [23,24].

According to Liu et al., the loss of PTEN suppression plus PI3K hyperexpression promotes cell proliferation, while its hypoexpression will promote cell differentiation [25]. For Richmond et al., PTEN is an important modulator of dormant stem cells, being regulated against stress or aggression stimuli, as represented by IR, in the present study [26].

In this context, after the genes analyzed in this study, we can suggest that the effect of combined therapy may be paradoxical on NS, therefore, according to our results, there was a dominant cytotoxic antineoplastic action by TMZ, and we observed an induction for expression of oncogenes in NS undergoing IR.

Following that way, Slaby et al. state that methylated (non-wild) MGMT is a predictive factor of response, especially for TMZ, but not among those undergoing combined TMZ + IR treatment. One possible explanation for radioresistance is based on the existence of miRNA-modulated glioma neural stem cells, which lose their quiescence and become indifferent according to their microenvironment and inductive stimuli [27].

In another study on miR-128 and miR-149 expression, She et al. demonstrated that these overexpressed miRNAs inhibit GBM invasion and increased their sensitivity to TMZ treatment [28]. In a study by Pan et al., the authors reduced proliferative and invasive activities in the U251 lineage of GBM, through the induction of inhibition of AKT, PCNA, CyclinD1, and MMP-2, via miR-149 [29]. This represents a potential anti-GBM cytostatic therapeutic target as it induced a pause at the G0 / G1 moment of mitosis [29].

According to Morgado et al., cell hypoxia injuries alter the GBM stem cells microenvironment, interrupting its quiescence and favoring its differentiation and invasion into adjacent tissue, and one of the miRNAs implicated in this process is miR-145, which becomes overexpressed, reducing the Sox2 and Lin28/let7 pathway, besides increasing the expression of let-7a and let-7b in process of gliomagenesis in GBM stem cells [30].

After this contextualization, in summary, our results show that in the T98G cell line there was increased expression of the MGMT, IDH1/2, EGFR, and PTEN genes and miR-181b, -145, -149, and -128a only when the NS were submitted to the combined intervention modality TMZ + IR. Thus, it is suggested that these miRNAs may be implicated with increased expression of these genes in the NS, when subjected to TMZ + IR, by inhibiting their counterregulatory. 

In the U343 cell line, we observed a higher expression of the genes MGMT, EGFR, and PTEN and miRNAs -145, -149, and -128a, which had their expression enhanced when the NS were submitted to IR alone. In this context, it is suggested that IR is an independent and determinant factor for NS radioresistance while being an effective treatment for adhered cells. Otherwise, we observed no downregulation complementarity action of the analyzed oncomiRs on the expression of the analyzed genes.

In this sense, while the Stupp regimen is more effective, it was hypothesized that sequential treatment initiated with isolated TMZ followed by TMZ + IR may be better than the current one, especially considering GBM stem cells as a target for treatment [19].

## Conclusions

Increased expression of the genes and miRNAs analyzed was observed in both the U343 and T98G cell lines, especially in the NS, when subjected to IR and TMZ + IR, respectively. Thus, it is suggested that IR determine the potential inducing effect of loss of quiescence in the NS and stimulate radio and chemoresistance. No restricted downregulation complementarity action of the analyzed oncomiRs on the expression of the studied genes was identified. Further studies on neurospheres are needed to understand the combined intervention modality (TMZ + IR).
